# Presumed Competent: The Strategic Adaptation of Asian Americans in Education and the Labor Market

**DOI:** 10.1146/annurev-soc-090523-051614

**Published:** 2024-02-05

**Authors:** Jennifer Lee, Kimberly Goyette, Xi Song, Yu Xie

**Affiliations:** 1 Department of Sociology, Columbia University, New York, NY, USA; 2 Department of Sociology, Temple University, Philadelphia, Pennsylvania, USA; 3 Department of Sociology, University of Pennsylvania, Philadelphia, Pennsylvania, USA; 4 Department of Sociology, Princeton University, Princeton, New Jersey, USA

**Keywords:** Asian Americans, education, labor market, bias, strategic adaptation, affirmative action, bamboo ceiling

## Abstract

Presumed competent, Asian Americans exhibit the highest level of education and median household income of all major US ethnoracial groups. On average, they outpace all groups in the domain of education, yet they do not maintain their advantage in the labor market. The question of bias against Asian Americans has taken center stage in the most recent US Supreme Court ruling on affirmative action, but the attention has been on university admissions. We broaden the focus and rewrite the question to consider how Asian Americans seek to preempt bias in the labor market by strategically adapting to mitigate it. Strategic adaptation begins with precollege education, continues with college choice and major, and entails acquiring elite credentials that signal hard skills and merit. The strategy falls short of obviating bias altogether, however. We show how Asian Americans’ labor market earnings and mobility vary by gender, nativity, national origin, place of education, and field of study.

## INTRODUCTION

Debates about merit, competence, and bias have emerged in unprecedented ways in the latest US Supreme Court (SCOTUS) ruling on affirmative action. The nonprofit organization, Students for Fair Admissions, charged Harvard University of violating Title VI of the Civil Rights Act by engaging in racial discrimination against Asian Americans. They maintained that Asian applicants are less likely to be admitted to Harvard than White, Black, and Hispanic applicants with comparable academic credentials. On June 29, 2023, SCOTUS ruled in favor of the plaintiffs and struck down race-based affirmative action in university admissions at both Harvard University and the University of North Carolina. Charges of bias against Asian Americans took center stage in the SCOTUS decision, but the focus has been narrowly limited to university admissions.

Asian Americans are not underrepresented in elite universities, however. Accounting for 7.2% of the US population, Asians comprise more than 20% of the undergraduate student body at Ivy League universities, including Harvard, whose most recent incoming class is 29.9% Asian (Amponsah & Haidar 2023). By nearly all measures, Asian Americans, on average, exhibit stronger educational outcomes than all other major US ethnoracial groups. They attain the highest grades in math and reading from kindergarten to high school (Hsin & Xie 2014), have higher standardized test scores, and enroll in more advanced classes (Caplan et al. 1991, Hsia 1988, Kao 1995, Sanchirico 1991, Zhou & Bankston 1998). In addition, Asian American students are less likely to repeat grades, more likely to complete high school, and also more likely to earn bachelor’s and postgraduate degrees (Xie & Goyette 2004).

In the United States,61% of Asian American adults have attained a bachelor’s degree compared with 42% of Americans identified as non-Hispanic White, 28% as African American/Black, and 21% as Hispanic/Latino (US Census Bur. 2022). While Asian Americans outpace all groups in education, they do not maintain their advantage in the labor market. They are the least likely to be promoted into leadership positions, even in fields in which they are overrepresented such as science, technology, engineering, and mathematics (STEM) and law.

Studies of bias against Asian Americans have focused on cultural differences, racial stereotypes, and institutional actors (including admissions officers, teachers, and employers), as well as the evaluation criteria they utilize. This body of research has examined how decision makers assess and respond to Asian Americans rather than how Asian Americans anticipate and respond to bias. In this review, we broaden the focus and rewrite the question to consider how Asian Americans seek to preempt bias by strategically adapting to mitigate it. Their strategic adaption involves acquiring elite credentials to signal hard, marketable skills in the labor market, but it falls short of combatting bias altogether.

Building on an earlier review by Sakamoto et al. (2009), we begin ours by presenting a portrait of the US Asian population, focusing on its rapid growth and diversity. The only ethnoracial group that is majority foreign born, Asian Americans are also the only group that is growing mainly by immigration. We then introduce the framework of strategic adaptation—coined by Xie & Goyette (2003)—to explain how a predominantly hyperselected, foreign-born group adapts to the labor market by choosing occupations in fields that require the most education and convey competence. Strategic adaptation begins well before labor market entry, however; it begins at earlier stages of the educational journey.

We argue that strategic adaption entails adopting and reinforcing a success frame that guides academic achievement, college choice, and college major in pursuit of a narrow set of highly credentialed professional occupations (Lee & Zhou 2015). These occupations are filled with the largest percentages of occupants with bachelor’s degrees or higher, such as the physical sciences, medicine, engineering, computer science, and law. Coupled with resources, this strategy yields an educational advantage over all other groups, but the advantage slips away in professional schools and the labor market.

Then, shifting to Asian Americans’ performance in law and business schools as well as in the labor market, we consider how college-educated Asian Americans fare compared with native-born Whites. Despite their exceptional educational attainment, Asian Americans do not retain an advantage (Chung et al. 2017; Gee & Peck 2017; Kim & Zhao 2014; Lu et al. 2020, 2022; Tran et al. 2019). While US-educated Asians have, on average, reached earnings parity with Whites, many also experience blocked mobility in the form of a so-called bamboo ceiling—akin to the glass ceiling for women. Except for US-born South Asian men, Asian Americans are the least likely group to be promoted into leadership positions in spite of their education, work experience, and job performance (Lu et al. 2020, 2022; Lu 2024; Xie 2022). We show how labor market outcomes—including earnings and career mobility—vary by gender, nativity, national origin, place of education, and field of study, and we also consider how native-born South Asian men have successfully broken through the bamboo ceiling. We conclude by pointing to fruitful directions for future research.

## THE GROWTH AND DIVERSITY OF ASIAN AMERICANS

The fastest growing group in the United States, Asian Americans increased from 0.6% of the county’s population in 1960 to 7.2% today. Between 2000 and 2019 alone, the US Asian population grew by 81% (Shimkhada & Ponce 2022). Unlike the demographic growth patterns of other groups including Hispanics, which are mainly driven by births, the US Asian population is growing primarily through immigration (Lee & Ramakrishnan 2020). The surge in immigration, accompanied by the lower birth rate of US Asians, has resulted in a population that is majority foreign born: Two in three Asian Americans are immigrants, a figure that increases to nearly four in five among Asian adults, and nine in ten are either immigrants or the children of immigrants. Asians stand out as the only major ethnoracial group in which a majority are born outside the United States—a fact that belies stereotypes and misperceptions of Hispanics as the quintessential immigrant group. By 2055, Asians are projected to surpass Hispanics as the largest immigrant group in the country (Colby & Ortman 2015).

### From Legacies of Exclusion to the Hyperselectivity of Asian Immigration

Despite the presence of Asian immigrants in the United States since the nineteenth century, the Asian population remained at less than 1% of the country’s total even as late as 1970. The Page Act of 1875, the Chinese Exclusion Act of 1882, the Gentlemen’s Agreement with Japan in 1907, and a series of other highly restrictive Asian exclusion laws lasting for six decades prevented the growth of the US Asian population (Lew-Williams 2018; Ngai 2004, 2021). Not until the 1965 Immigration and Nationality Act did the doors to the United States open to many Asian immigrants. Abolishing the national-origin quotas that suppressed Asian immigration for decades, the 1965 Immigration and Nationality Act created new preferences for foreign-born applicants based on family reunification, professional skills, and refugee status. As a result of these changes, the earliest post-1965 Asian immigrants were highly educated, highly selected, and professionally skilled, including doctors, nurses, and engineers.

The high immigrant selectivity of contemporary Asian immigration is one of its defining characteristics. Indeed, so highly selected are immigrants from China, India, the Philippines, and Korea that Lee & Zhou (2015) characterize them as hyperselected to reflect their dual positive selectivity: They are not only more likely to have graduated from college than their nonmigrant counterparts from their countries of origin but also more likely to hold a college degree than US residents. Indians are the most highly educated: 79% of Indian immigrants in the United States hold a BA or higher compared with only 8% of India’s population, indicating that US Indian immigrants are 10 times more likely to be college educated than their nonmigrant counterparts. Among the five largest US Asian groups—Chinese, Indians, Filipinos, Vietnamese, and Koreans—all but Vietnamese (the only refugee group) are hyperselected (Tran et al. 2019). These top five Asian groups account for 83% of the US Asian population.

Groups such as Vietnamese, Cambodians, Hmong, and Laotians began arriving en masse as refugees in the late 1970s (Hein 2006, Zhou & Bankston 1998). Fleeing turbulent political regimes, these refugee groups display a range of socioeconomic profiles, with the first wave typically highly educated and hyperselected and later waves significantly less so. Not only did their contexts of exit differ from those of voluntary immigrants like Chinese, Japanese, and Koreans, but so too did their context of reception (Portes & Rumbaut 2006); the US government provided various forms of resettlement assistance to help ease the incorporation of war-torn refugees.

Today’s newest Asian immigrants are increasingly from South Asia—with origins in India, Pakistan, and Bangladesh—and exhibit an array of skills and education levels. Indians are among the most highly educated and hyperselected immigrants. Bangladeshis, by contrast, fall at the lower end of the socioeconomic distribution, in regard to both educational attainment and income.

The shift in national origins among Asian immigrants has resulted in unprecedented diversity within the US Asian population (Drouhot & Garip 2021, Lee et al. 2018, Lee & Ramakrishnan 2020, Wong & Shah 2021, Xie & Goyette 2004). Historically, the majority of Asian immigrants in the United States were East Asian, but current trends show a more balanced distribution across various Asian regions. South and Southeast Asians now comprise 29% and 32% of the Asian American population, respectively, while East Asians account for 34% (Ramakrishnan 2023).

In spite of their diversity, Asian Americans are, on average, highly educated. Their remarkable educational attainment is, in part, a consequence of migration selection. Hyperselectivity provides Asian immigrants and their US-born children with economic, ethnic, and social psychological advantages over hyposelected immigrant groups like Mexicans (Diaz & Lee 2020, Fernández-Kelly 2020, Telles & Ortiz 2009), as well as native-born Whites and Blacks, in the domain of education (Fernández-Kelly 2016, Goyette & Xie 1999, Hsin 2016, Hsin & Xie 2014, Lee & Sheng 2024, Lee & Zhou 2017, Tian 2023).

Hyperselected immigrants are more likely to set high educational goals for their children and more likely to provide the resources to achieve them, such as supplemental education, SAT prep courses, tutoring, and extracurricular activities (Goyette & Xie 1999, Lee & Zhou 2015, Tian 2023, Warikoo 2022). Yet hyperselectivity alone does not fully account for Asian Americans’ educational advantage. Asian Americans’ high educational aspirations and education-related investments are much less differentiated by family socioeconomic status (SES) (Fishman 2020, Goyette & Xie 1999, Liu & Xie 2016, Tian 2023). Beliefs about effort are also critical. Research reveals that Asian American families tend to hold a stronger belief in the connection between effort and educational success than White families (Chen & Stevenson 1995, Liu & Xie 2016). As a result, Asian American students exhibit greater effort in their schoolwork, thereby yielding stronger academic outcomes than native-born Whites (Hsin & Xie 2014, Jiménez & Horowitz 2013).

### Stereotypes of Hyperselected Asian Americans and Their Consequences

Asian Americans—regardless of nativity and national origin—are racialized similarly in the US context and subject to stereotypes about their perceived competence and lack of sociability and warmth (Lee & Fiske 2006, Lin et al. 2005, Zhang 2015, Zou & Cheryan 2017). In schools, Asian Americans are often deemed by their teachers, guidance counselors, and peers as diligent, hardworking, and suitable for competitive academic tracks (Drake 2022, Musto 2019). As Lee & Zhou (2015) have shown, this can lead to stereotype promise—the boost in performance that comes with being perceived through the lens of a positive stereotype.

Other research points to how positive perceptions of Asian students can affect teachers’ evaluations of even their non-Asian peers. Shi & Zhu (2023) find that teachers over-rate Asian students in their evaluations compared with White students with comparable standardized test scores—even after adjusting for nativity, class, and a host of measures like school absences. They also find that the presence of Asian students has spillover effects for Black and Hispanic students: A single Asian student in a classroom amplifies teachers’ negative assessments of Black and Hispanic students relative to academically comparable White students.

Critically, Shi & Zhu (2023) find that teachers’ positive bias toward Asian students is stronger than their negative bias toward Black and Hispanic students. This finding is especially noteworthy considering the large body of research that shows how stereotype threat—the anticipation that one’s performance will be judged according to their membership in a particular ethnoracial group—can undermine the academic performance of otherwise high-achieving Black students (Steele & Aronson 1995). By comparison, there has been relatively little research on the potential positive biases that can enhance the academic performance of otherwise mediocre Asian American students (Shih et al. 1999), including stereotype promise (Lee & Zhou 2015, Okura 2022).

The intersection of race, gender, and nativity produces a unique set of stereotypes. East Asian men—regardless of nativity—are described as shy, diffident, passive, uncreative, socially distant, and lacking in confidence (Chin 2020, Kao et al. 2018, Lu 2024), whereas foreign-born South Asian men are considered hostile and aggressive (Chavez 2021). Both US- and foreign-born Asian American women are believed to be docile, submissive, hyperfeminine, and the least fit to assume leadership posts, in spite of their experience, performance, or behavior (Tinkler et al. 2019).

While stereotypes of Asian Americans’ hard work and diligence may work in their favor in elementary and secondary schools, the same set of stereotypes may hamper them in university admissions and the labor market. Presumed competent, Asian Americans are considered mathematically and scientifically inclined, but not artistically nor verbally gifted (Chou & Feagin 2015, Lee 2008, Lee & Fiske 2006, Leong & Hayes 1990, Lin et al. 2005, Tan 1994, Tuan 1998). Competence can also be a curse (Lee 2007): Technically strong but socially weak, Asian Americans may be recognized for their dedication and effort but are not considered to be innately brilliant, creative, charismatic, or fit to lead (Lu 2024, Lu et al. 2020).

In the case of *Students for Fair Admissions, Inc. v. President and Fellows of Harvard College* (2023), the plaintiffs charged that the university employed these racial stereotypes to poorly rate Asian American applicants’ “personal qualities” in order to depress their chances of admission (Lee 2021). While accusations of bias against Asian Americans in university admissions reached SCOTUS, questions of bias in the workplace have received relatively scant attention. The inattention is glaring given the well-documented pattern of Asian Americans’ (with the exception of South Asians’) blocked career mobility into management and executive positions.

Below, we shift the focus and synthesize the research on Asian Americans in education and the labor market in a novel way to show how college-educated Asian Americans attempt to preempt bias in the labor market by strategically adapting to it (Xie & Goyette 2003). We stress that strategic adaptation begins early on with Asian American families’ decisions about primary education and continues through higher education and in the labor market.

## THE STRATEGIC ADAPTATION OF ASIAN AMERICANS

The roots of strategic adaptation—a framework coined by Xie & Goyette (2003)—are found in earlier research by Sue & Okazaki (1990) and Kao et al. (1996). Due to blocked opportunities for social mobility, Asian Americans pursue career options that are most likely to shield them from societal bias. Educational attainment is foundational to this strategy.

Because education is perceived to be an institution that should reward individuals based solely on ability and hard work, rather than on “functionally irrelevant” (Cole & Cole 1973) characteristics such as race, gender, and SES, researchers find that Asian Americans perceive education as the surest path to attaining prestigious, high-earning careers. This perception is bolstered by historic connections between educational attainment and socially prestigious careers in traditionally Confucian countries, as well as the contemporary emphasis on education as the single most important engine of mobility in East Asian countries like China, Japan, and Korea. These perceptions are especially acute among the foreign born, who comprise the majority of Asian Americans.

Sue & Okazaki (1990) propose that Asian immigrant parents—realizing their US-born and -raised children may encounter barriers to social mobility—stress education as a means to overcome obstacles. They refer to this response to blocked mobility as “relative functionalism.” Parents emphasize and prioritize academic achievement for their children in order to compensate for their limited political resources, lack of mainstream social networks, societal bias, and institutional discrimination. Asian immigrant parents focus on education, in particular, because of their strong belief in the connection between hard work and success (Chen & Stevenson 1995, Hsin & Xie 2014), which underlies their investment in education as a means to overcome potential challenges to future mobility.

Xie & Goyette (2003) elaborate on the concept of relative functionalism with their framework of strategic adaptation. They argue that Asian Americans strategically choose fields that have high educational requirements, such as the physical sciences, medicine, engineering, and computer science, to ensure a smoother path to mobility. Notably, these fields have the highest percentages of occupants with bachelor’s degrees.

Since this earlier work, more research has examined the ways that Asian American individuals and their families strategically plan to ensure both educational and career success (Dhingra 2020, Huang 2021, Lee & Zhou 2015, Tian 2023, Warikoo 2022). While not all of the research explicitly identifies Asian Americans as being deliberately strategic, we argue that taken together, this body of work shows how Asian American individuals and families make decisions that enhance their own and their children’s educational prospects with an eye toward their future career outcomes.

We present an overview of research indicating that Asian American families prioritize neighborhoods with high-performing schools for their children and are deliberate about their children’s educational expenditures and extracurricular activities. Asian American students also make strategic decisions about college and field of study. They are more likely to apply to and attend selective, reach colleges. Once enrolled, they are more likely to major in business, STEM, and medical fields. Such strategic decisions often lead college-educated Asian Americans into high-earning careers, as we elaborate below.

### Housing and School Choice

First, Asian American parents are strategic in their choice of elementary and secondary schools for their children, with an eye toward selecting neighborhoods with high-performing public schools (Lee & Zhou 2015, Park 2020, Warikoo 2022). To this end, Asian American families with K–12-aged children spend more on housing than do comparable White families to have access to more competitive, higher quality schools (Tian 2023). While many of these schools are in predominantly White suburban communities, the rise of middle-class Asian American “ethnoburbs”—particularly in California, New York, and New Jersey—has led to high concentrations of Asian American students in public schools that boast high average test scores and high rates of attendance in four-year colleges (Drake 2022, Huang 2010, Kye 2018, Li 1999). Because of this, Asian American students may find themselves in an educational environment surrounded by other high-performing Asian peers (Kim & Gasman 2011, Massé 2019), including those from educationally competitive East Asian countries like China and Korea who complete high school in the United States, often migrating with their mothers or alone (Kim & Okazaki 2014, Koo & Lee 2006).

While Asian American students are not more likely to attend private schools (Goyette & Xie 1999), they are significantly more likely to attend selective public high schools. They make up a disproportionately large share of students in nationally renowned public schools such as Stuyvesant, Bronx High School of Science, and Lowell High School. At Stuyvesant, for example, Asian American students comprise 72% of the student body, while Asians account for only 12% of New York City’s population. At Lowell High School, Asian American students account for 57% of the student body, while Asians account for 37% of San Francisco’s population. The severe underrepresentation of Black and Hispanic students in these selective public schools has spawned heated debates about opportunity, merit, exclusion, and fair admissions in cities like New York, San Francisco, Chicago, and Philadelphia (Allensworth & Rosenkranz 2000, Chuang 2001).

### Educational Expenditures and Extracurricular Activities

Second, Asian American families are strategic about their children’s educational expenditures, including extracurricular activities during primary and secondary school, even after adjusting for SES. Tian (2023) finds that Asian American families spend more on extracurricular activities overall, particularly on academic and enrichment activities, than do White families with similar socioeconomic backgrounds. Where Asian families spend less than their White counterparts, however, is on sports and recreational activities. Other research shows that East Asian students engage in test prep and other private supplemental academic programs like Kumon and Sylvan Academies more so than their peers of other ethnoracial backgrounds (Byun & Park 2012, Park et al. 2016), while South Asian children turn to spelling bee competitions to enhance their skills (Dhingra 2020, Shankar 2019).

Unlike Black and Hispanic students who use supplemental education to catch up, Asian students leverage it to secure a competitive edge or to get ahead (Ho et al. 2019). Furthermore, compared with White families, Asian American families tend to stress the competitive aspects of education, often encouraging competition in grades, test scores, honors, and extracurricular contests. In part, this stems from the belief among Asian American parents that their children will be primarily assessed in comparison more with other high-achieving Asian American students during college admissions than with their White peers (Dhingra 2020, Tian 2023, Warikoo 2022).

In addition, because hyperselected Asian immigrant groups like Chinese and Koreans create educational services in their communities—sometimes at low or no cost in community centers, ethnic churches, and temples—supplemental education becomes accessible to coethnics across class lines (Lee & Zhou 2015, 2017; Zhou & Kim 2016). Even during the COVID-19 pandemic, supplemental education did not come to a halt for Asian American students (Lee et al. 2023). South Asian students focused on test preparation and other online supplemental services, while East Asian students were more likely to get private tutoring compared with their White peers.

### Applying to Selective and Reach Institutions

Third, relative to White students, Asian American high school students not only are more likely to apply to and enroll in college, but they are the most likely to apply to and enroll in selective and reach colleges and universities (An 2010, Ayalon et al. 2008, Carnevale & Rose 2004, Fishman 2020). While one might attribute this difference to Asian American students having, on average, higher GPAs and SAT or ACT test scores compared with other students, even after controlling for these indicators of academic achievement, Asian Americans are still more likely to attend selective institutions (Mullen & Goyette 2019). Looking from a different angle, researchers have found that Asian American students are also more likely than White students to apply to reach institutions—defined as institutions for which students’ own SAT or ACT scores are in the 25th percentile for the overall student body (Fishman 2020, Mullen & Goyette 2019). These patterns suggest that Asian Americans are more likely to seek to maximize the prestige of their college degree relative to their qualifications compared with other groups (Fishman 2020, Goyette et al. 2024).

### Financial Contributions to Children’s Higher Education

Fourth, Asian American families tend to save more and invest more in their children’s education—including supplemental education—than other ethnoracial groups. This may be because they perceive the higher cost of attending a selective university to be worth the return (Dondero & Humphries 2016, Goyette et al. 2024, Ouyang et al. 2019). Research confirms the multidimensional rewards of attending a selective university: They boast higher graduation rates (Long 2008), they are associated with higher earnings later in graduates’ careers (Brand & Halaby 2006, Thomas & Zhang 2005, Witteveen & Attewell 2017), and they garner greater esteem from students’ peers and families’ social networks.

### College Major Choice

Finally, strategic adaptation continues through college. Once enrolled, Asian American college students are more likely to choose majors that lead to high-paying careers, such as physical sciences, engineering, computer science, and business (Goyette & Mullen 2006, Xie & Goyette 2003). This is particularly the case among students who attend four-year colleges (Ma 2009). Self-stereotyping may also affect the choice of college majors for Asian American students: Those who believe stereotypes that Asian American students are academically talented yet less skilled in English tend to gravitate toward math and science majors (Shen 2015). While there is some evidence that the high concentration of Asian American majors in STEM and medical fields declines over immigrant generations for some groups (Min & Jang 2015), it remains higher than for US-born White students.

Asian American students choose fields with higher starting salaries than their White peers (Alba & Barbosa 2016), but significant differences between Asian American men (with the exception of Southeast Asians) and White men disappear after accounting for their likelihood of attending college (Song & Glick 2004). The same does not hold true for Asian American and White women: Chinese, Filipino, and Southeast Asian women choose fields associated with more lucrative careers than do White women (Song & Glick 2004).

In sum, attending selective, reach undergraduate institutions is an integral component of Asian Americans’ strategic adaption: Elite institutions are more assured vehicles to high-status, high-earning careers in the face of scarce social networks, limited political and cultural capital, and stereotypes that racialize Asian Americans as competent but neither brilliant nor warm. Elite educational credentials become a means of achieving mobility in the face of anticipated racial bias. But a new vein of research shows the limits of strategic adaptation, which become apparent in professional schools like law and business, as we show in the section that follows.

### A Bamboo Ceiling in the Classroom

The educational advantage that Asian American students exhibit from elementary to high school and through college begins to disappear in professional graduate schools, pointing to what researchers suggest may be a “bamboo ceiling in the classroom” (Lu et al. 2022, Xie 2022). In law and business school, East Asians—but not South Asians—attain lower grades than their White peers, even after controlling for overall standardized test scores, nativity, and school-level characteristics. East Asian students receive poorer grades in leadership, strategy, and marketing courses in which class participation is a larger percentage of the final grade. Differences in verbal assertiveness—rather than ability or motivation—as Lu et al. (2022) argue, are where East Asian students fall short, which, in turn, explains their lower grades.

Buttressing their thesis is the additional finding that the East Asian disadvantage in the classroom was muted in online Zoom courses. In spite of the same content covered in both in-person and online classes, East Asian students did not receive poorer grades in courses taught online. Lu et al. (2022) speculate that social presence is less salient in online classes, so that East Asian students are less disadvantaged relative to other groups in teachers’ evaluations for classroom participation.

While Lu et al. (2022) analyzed complete cohorts of MBA and JD students, Xie (2022) cautions that their sample was selected on an outcome variable—current enrollment of students in these professional schools—which may create “collider bias” (Morgan & Winship 2015). Since admission committees typically consider a combination of factors to create a cohort, one factor (such as quantitative test scores) may be negatively correlated with another (such as leadership potential) in the sample of admitted students. Since the East Asian students in Lu et al.’s (2022) sample were stronger in their quantitative test scores, they may be weaker in leadership potential, even if these variables are unrelated in the general US population (Xie 2022).

Xie (2022) also points out that while Lu et al. (2022) controlled for students’ nativity in their analyses, they did not control for the place of undergraduate education among their students. This oversight is critical since Zeng & Xie (2004) have shown that among foreign-born Asians, those who attain their highest educational degree abroad experience an earnings disadvantage in the labor market relative to Whites as well as US-born and US-educated Asians. Since Lu et al. (2022) did not assess variation in the place of education among East Asian students, foreign undergraduate education—rather than verbal assertiveness alone—may account for at least some of the East Asian disadvantage in the classroom, especially given the lower verbal test scores (but not lower quantitative scores) of the East Asian international students in their sample.

## ASIAN AMERICANS’ LABOR MARKET (DIS)ADVANTAGE

While most studies examining Asian Americans’ educational outcomes point to their advantage, research on their labor market outcomes has yielded mixed evidence. Some research shows that US-born and -educated Asians have reached parity with Whites (Zeng & Xie 2004), while other research suggests that there are wage differences between Asians and Whites that are unexplained by traditional human capital theory (Chiswick 1983, Sharpe & Abdel-Ghany 2006). Still, other studies acknowledge parity between Asian Americans and Whites, but only because the former are overeducated vis-à-vis the latter (Hirschman & Wong 1984).

### The Overeducation Hypothesis

In a seminal study on SES attainment of Asian Americans, Hirschman & Wong (1984) adapted Blau & Duncan’s (1967) classic status attainment model to examine racial gaps in socioeconomic index (SEI) scores (Duncan 1961). The score—calculated as a weighted sum of the average education and average income of all workers in an occupation—is a hallmark of research on social stratification and mobility during the 1960s–1980s (Ganzeboom et al. 1991, Treiman & Ganzeboom 2000).

Asian Americans have reached socioeconomic parity with Whites, but Hirschman & Wong (1984) concluded that this is a product of Asian Americans’ educational overachievement. If Asians were subject to the same stratification process as Whites, their educational qualifications would significantly elevate their occupational attainment and earnings, thereby surpassing those of Whites. Hence, equality between Asians and Whites stems from the fact that Asians experienced unequal—specifically, lower—economic returns to education than Whites.

Since Hirschman & Wong (1984) proposed the overeducation hypothesis, it has been subject to numerous tests in subsequent studies using updated data, alternative methodologies, and outcomes beyond the SEI measure (Barringer et al. 1990; Greenman 2011; Iceland 1999; Sakamoto et al. 2009, 2012; Snipp & Hirschman 2005; Xie & Goyette 2003; Zeng & Xie 2004). New strands of research on Asian Americans’ labor market outcomes focus on nativity, place of education, field of study, gender, and blocked career mobility in the form of a bamboo ceiling.

### Nativity, Place of Education, and Field of Study

Given that four-fifths of Asian American adults are foreign born, Zeng & Xie (2004) test the overeducation hypothesis by considering how nativity and place of education may be potential confounding factors in Asian Americans’ earnings disadvantage. Focusing on Asian American men and drawing on individual-level data from the 1990 US Census and the 1993 National Survey of College Graduates, Zeng & Xie (2004) find no earnings differences among US-born Whites, US-born Asians, and US-educated Asian immigrants. The only group that experiences an earnings disadvantage is foreign-educated Asian immigrants. They conclude that once place of education is taken into account, race and nativity are inconsequential for college-educated US Asians’ earnings.

Other research confirms the place of education thesis (Arbeit & Warren 2013, Lu & Li 2021, Tong 2010). Tong (2010) finds that among immigrant scientists and engineers, attending college and/or precollege in the United States plays a substantial role in closing the earnings gap with their native-born counterparts. US-educated individuals not only have higher earnings but also experience a faster earnings growth rate compared with their foreign-educated counterparts. Notably, however, Tong (2010) did not control for field of study. Apart from earnings, place of education also affects the likelihood of attaining managerial positions: Foreign-born Asians who received either no US education or only higher education in the United States were less likely to be in managerial positions than native-born Whites, with the place of education more salient for East Asians than South Asians (Shao 2023).

However, other research points to an earnings disadvantage among US-born Asians, even after controlling for place of education. Analyzing cross-sectional data from the 2003 National Survey of College Graduates, Kim & Sakamoto (2010) find that after controlling for field of study, native-born, college-educated Asian men earn less than White men, evening after adjusting for their years of schooling, place of education, major, region of residence, parents’ education, and other demographic characteristics. Curiously, they also find that 1.5-generation Asian men (foreign-born Asians who migrated to the United States before the age of 13 and were educated in the United States) have reached full parity with their White male counterparts after controlling for region, field of study, and college type. Controlling for field of study, Kim & Sakamoto (2010) note, is critical in understanding the Asian–White earnings gap.

### Gender

Earnings disparities between Asians and Whites tend to be more consequential for men than for women (Browne & Misra 2003, Xie & Greenman 2008), including immigrant women (Tong 2010). Some research shows that native-born Asian American women are unique among non-White women in that they do not experience an earnings disadvantage compared with White women with similar levels of education (Greenman 2011, Iceland 1999). Iceland (1999) finds that native-born Asian women from different ethnic groups do not experience significant earnings disparities compared with similarly educated native-born non-Hispanic White women, while Greenman (2011) shows that Asian women have higher earnings than comparable White women. Analyzing data from the Scientists and Engineers Statistical Data System, Greenman (2011) finds that the advantage of Asian American women is attributable to their lower tendency to withdraw from the workforce after transitioning to motherhood. Because they are less likely to reduce their labor supply, Asian women accumulate more work experience than White women and therefore enjoy a higher rate of earnings growth over time.

Other research challenges the thesis of Asian American women’s advantage. Based on analyses of the 2003 National Survey of College Graduates, after controlling for field of study, college type, region of residence, and other demographic variables, Kim & Zhao (2014) find that Asian American women are more likely to be unemployed, and even when employed, they are less likely to attain positions that involve supervising a large number of people than White women. They also note differences among Asian American women: foreign-born Asian women who immigrated after high school are disadvantaged with respect to not only unemployment and management, but also earnings. Furthermore, this disadvantage holds even if Asian American women earned their highest degree at a US institution.

Some US education does help foreign-born Asian American women who immigrated before high school (the 1.5 generation); they reach parity with White women with respect to earnings and employment rates but remain disadvantaged in the number of people they supervise. Findings that highlight the lower likelihood of Asian American women and men ascending into managerial and executive positions have helped spawn a new vein of research on the bamboo ceiling.

### The Bamboo Ceiling and South Asian Exceptionalism

In *Breaking the Bamboo Ceiling*, career coach Jane Hyun (2005) pointed to the lack of Asian Americans in executive suite positions and coined the term bamboo ceiling to describe the barriers Asians face in advancing to top positions in organizations. Empirical evidence shows that despite their exceptional educational achievement, Asian Americans lag behind in promotion to managerial and executive ranks after adjusting for years of work experience and performance reviews (Chung et al. 2017, Fernandez 1998, Gee & Peck 2017). Even in fields in which Asian Americans are overrepresented, such as technology, law, medicine, the natural sciences, and engineering, they face the lowest rates of promotion compared with all other ethnoracial groups (Mervis 2005).

Recent reports of top technology firms in Silicon Valley show that White men and women are twice as likely as Asian men and women, respectively, to advance into the executive ranks (Gee & Peck 2017). A similar pattern emerges in law, in which Asians comprise 10% of graduates of the top 30 law schools, but only 6.5% of all federal judicial law clerks. And while Asians are the largest non-White group in major law firms, they have the highest attrition rates and the lowest ratio of associates to partners of all groups at 4 to 1, compared with 2 to 1 for Blacks and Hispanics and parity for Whites (Chung et al. 2017). Even in academia, where Asian Americans are overrepresented as students in elite universities, they are nearly absent in leadership ranks, comprising only 2% of college presidents.

Researchers have focused on the reasons for the bamboo ceiling and Asian Americans’ blocked intragenerational mobility—that is, the extent to which individuals can move to different socioeconomic positions, especially in terms of jobs, occupations, leadership roles, managerial positions, or other indicators of professional success during their adult lives (reviewed in Hollister 2011, Kalleberg & Mouw 2018, Rosenfeld 1992). Some argue that Asians’ advancement in the workplace is hindered, in part, by racial, gendered, and cultural stereotypes (Chin 2020). Presumed competent, Asian Americans are perceived as intelligent, educated, hardworking, and diligent (STAATUS Index 2023), but not visionary, creative, or socially adept enough to assume leadership positions (Lu 2024). Other studies suggest job mismatch—in terms of educational qualifications, skills, and field of study—as a mechanism to explain Asians’ wage disadvantage, underemployment, and job displacement (De Jong & Madamba 2001, Lu & Li 2021, Madamba & De Jong 1997). In addition, there is a double disadvantage for Asian American women, who are the least likely group to be promoted to leadership and also the least likely to be perceived as fit for leadership roles regardless of their experience and behavior (Tinkler et al. 2019).

Other research by Lu et al. (2020, 2022) points to differences in culture and, more specifically, differences in verbal assertiveness between East Asians and Whites. In Western corporate culture, assertiveness conveys competence, confidence, creativity, and charisma. The clash between a US corporate culture that rewards individual displays of distinction and East Asian Confucian values that promote harmony and interpersonal relationships may be at the root of the bamboo ceiling for East Asian professionals. Buttressing their thesis, they also find that South Asians (whose roots are not Confucian) are verbally assertive—both in the workplace and in the classroom—and they excel in both domains. In fact, South Asian men are more likely than White men to attain leadership positions (Lu et al. 2020)—pointing to a pattern of South Asian exceptionalism.

The pattern of South Asian exceptionalism may also be explained by Americans’ understanding of who counts as Asian (Goh & McCue 2021, Lee & Ramakrishnan 2020). In the US context, the default characterization of “Asian” is East Asian, and most Americans—including most Asian Americans—exclude South Asians from the fold (Lee & Ramakrishnan 2020). As a result, South Asians may not be perceived through the same stereotypical lens as East Asians, which may affect not only the behavior of South Asians but also the ways in which their behavior is interpreted by others in the workplace. If the stereotypical perception of Asian men (i.e., East Asian men) is that they are shy, diffident, and socially distant, South Asian men (who are not perceived as Asian) may not be hampered by a social identity that presumes these qualities.

In spite of recent studies that point to blocked intragenerational mobility, there is also evidence that challenges the bamboo ceiling for US-born Asians at the managerial level. In the government sector, for example, Sakamoto et al. (2006) find that native-born Asian American men are at least as likely to be employed as managers as are White men after adjusting for education and other demographic variables. Moreover, in the non-self-employed private sector, they find only limited evidence of a bamboo ceiling. They conclude that while there may be differences in managerial authority that their data cannot capture, the bamboo ceiling is not as pervasive as other studies suggest.

## DIRECTIONS FOR FUTURE RESEARCH

Research on Asian Americans has focused disproportionately on their exceptional educational achievement. In spite of social scientists’ explanations of these patterns, the narrow focus on education has had the unintended consequence of reifying the perception that Asian Americans are the advantaged minority or the so-called model minority. Future research should move beyond education and into the labor market, where Asian Americans no longer maintain an advantage and where evidence of their outcomes is more mixed.

While there is growing literature on Asian Americans’ earnings, due to data limitations, these studies primarily rely on a snapshot or single-point-in-time measures rather than life-course earnings trajectories. Given the depth of research on long-term earnings and wage and wealth gaps among non-Hispanic Whites, Blacks, and Hispanics (Cheng 2016, Cheng et al. 2019, Chetty et al. 2020, Collins & Wanamaker 2022, Killewald et al. 2017, Song et al. 2022, Tomaskovic-Devey et al. 2005), research that focuses on Asian Americans’ labor market attainment and inequality from a longitudinal perspective is sorely lacking. Longitudinal data such as those used by Greenman (2011) in her study would enable researchers to decompose earnings gaps between Asian American, White, and non-White workers into initial earnings positions in the entry-level job market as well as cumulative earnings growth over time (Cheng & Song 2019, Xie & Gough 2011). Given that Asian Americans often constitute a small fraction of the sample in social surveys or are altogether excluded, administrative data might offer significant insights for analysis (Song & Coleman 2020).

Moreover, research to date has not developed a dynamic view of long-term occupational movement among Asian Americans in comparison to other groups. Existing studies of the in-tragenerational mobility of Asians draw predominantly on cross-sectional data, focusing on predicting the unequal chances of job promotion across ethnoracial groups. On the other hand, recent reports provide a wealth of descriptive data on the severe lack of Asian Americans in leadership positions. To advance the field of Asian American attainment and mobility, investment in longitudinal data is critical for researchers to develop transition or network models to explain Asian Americans’ occupational mobility, akin to those designed to understand Black–White differences ( Jarvis & Song 2017, Parrado et al. 2007, Tomaskovic-Devey et al. 2005, Villarreal 2020).

In addition, most studies of Asian Americans’ earnings and career mobility have focused predominantly on Asian male workers due to the small sample size of Asian female workers who have been consistently employed in the labor market. This single-gender perspective offers an incomplete portrait of Asian Americans’ labor market outcomes. Gender may play an important role in shaping racial inequality between Asian Americans and other groups, as reports consistently show that college-educated Asian American women are the only non-White group that does not experience an earnings disadvantage compared with White women. Despite this, Asian women also represent the demographic group least likely to be promoted into leadership positions. Studying Asian Americans’ outcomes from an intersectional perspective constitutes a promising direction for future research (Vo et al. 2023).

Apart from gender, we suggest that researchers continue to disaggregate the Asian category in their analyses given the diversity of the US Asian population (Lee & Ramakrishnan 2021, 2020; Xie & Goyette 2004). While studies have shown that race and national origin may not be the first-order cause of Asians’ (dis)advantage in the labor market, most prior research did not disaggregate the Asian category. Thus, the SES of Asian Americans can be characterized best by a “high average and a large dispersion” (Zeng & Xie 2004, p. 1076).

Data disaggregation is critical to accurately studying the outcomes of US Asians, who number over 24 million in the 2020 Census and hail from more than 20 countries in East, Southeast, and South Asia. Past research reveals differences between East and South Asian workers—pointing to communication styles in particular—that affect labor-market rewards (Lu et al. 2020). Other work focuses on differences in immigrant generation, showing that foreign-born Asian workers experience more labor-market disadvantage than their US-born counterparts (Kim & Sakamoto 2010), but only for those who attended college outside the United States (Zeng & Xie 2004). Only with disaggregated data will researchers be able to study the differences within the Asian American population, which are often greater than the differences between Asians and other US ethnoracial groups (Lee et al. 2018, Vo et al. 2023).

Apart from advancing research through longitudinal perspectives and disaggregating Asian Americans by gender, nativity, place of education, field of study, and national origin, we also suggest that researchers study the role of assertiveness in labor market outcomes, including but not limited to promotion to managerial and executive ranks. While Lu et al. (2020, 2022) find that being less assertive may be detrimental to attaining leadership positions in the United States, Xie (2022) posits that it may actually promote high socioeconomic attainment more generally. For example, East Asians may be more likely to believe in the importance of effort—that is, one should be well-prepared and thorough before one speaks up. Communication—especially with authority figures—should reflect what comes from one’s best effort rather than one’s initial impulse for the sake of verbal assertiveness. In other words, what is considered a disadvantage by Lu et al. (2020, 2022) for leadership positions may also be an advantage for East Asians in more general settings where evaluation depends on hard skills and concrete criteria, such as a test, a paper, a report, meeting deadlines, or job performance in general, in which improvement through effort matters and pays off.

We would be remiss if we did not point out that future research should continue to examine how the geopolitical threat of China affects Asian Americans’ career trajectories, and those of Chinese Americans in particular (He & Xie 2022, Lee 2022). After President Trump launched the China Initiative in 2018 to thwart espionage by the Chinese government, more than two dozen academic scientists, most of whom were of Chinese origin, faced federal charges. Despite failed prosecutions, the accusations and lengthy investigations have upended the careers of many of these accomplished scientists. After 4 years of investigations, the Department of Justice—under a new administration—announced in 2022 that the China Initiative would come to a formal end. But the China Initiative has already produced a chilling effect on US–China scientific collaborations and has generated feelings of fear and anxiety among Chinese-origin scientists in the United States. Not only are scientists of Chinese origin less likely to apply for federal grants, but some are even considering leaving the United States (Xie et al. 2023). The impact of geopolitical relations and tensions on the career choices and trajectories of Chinese Americans in particular is critical to study.

While Asian Americans are the fastest growing and most diverse ethnoracial group in the United States, their experiences are nearly absent in university curricula (Lee & Huang 2021), often overlooked in research, and missing from data collection efforts. This bias has resulted in the systematic invisibility of Asian Americans in sociological research, curricula, and funding priorities. Of the $19 billion awarded by private foundations between 1983 and 1990, for example, only 0.18% was awarded to Asian American and Pacific Islander (AAPI) organizations (Ramakrishnan et al. 2020). In 2018, this increased to 0.20%. Another way to think about this is for every $100 awarded by foundations, only 20 cents was designated for AAPI communities. Similarly, the share of the total National Institutes of Health budget allocated for clinical research that focused on Asian American, Native Hawaiian, and Pacific Islander populations rose marginally, from 0.12% between 1992 and 2000 to 0.18% post-2000 (Đoàn et al. 2019). Despite these incremental increases, research that focuses on Asian Americans remains woefully underfunded. We cannot understand race, which is so central to the American social structure, and indeed, the whole American project, if we do not incorporate the study of Asian Americans in our funding priorities and our research.

We also urge researchers to move past the domain of education and into the labor market— where Asian Americans’ strategic adaptation carries them only so far—and also consider different methods to study the variegated approaches that Asian Americans employ in their life courses as they attempt to preempt bias. Moreover, attention should be paid to both ends of the stratification order in the labor market—highly educated professionals on the one hand and unskilled laborers and service workers on the other. Studying the labor market outcomes of Asian Americans carries significant implications for US ethnoracial relations at a time when race-conscious affirmative action in higher education has been dismantled, anti-Asian violence and hate have surged since the onset of COVID-19, and the geopolitical threat of China continues to loom. This research will help inform individuals, businesses, and policymakers on identifying and removing race-based barriers to mobility, thereby providing more equal opportunities not only for Asian Americans, but for all US workers.
